# Hypoxic Conditioning as a New Therapeutic Modality

**DOI:** 10.3389/fped.2015.00058

**Published:** 2015-06-22

**Authors:** Samuel Verges, Samarmar Chacaroun, Diane Godin-Ribuot, Sébastien Baillieul

**Affiliations:** ^1^Laboratoire HP2, Université Grenoble Alpes, Grenoble, France; ^2^U1042, INSERM, Grenoble, France

**Keywords:** intermittent hypoxia, conditioning, therapeutics, dose, humans, murines

## Abstract

Preconditioning refers to a procedure by which a single noxious stimulus below the threshold of damage is applied to the tissue in order to increase resistance to the same or even different noxious stimuli given above the threshold of damage. Hypoxic preconditioning relies on complex and active defenses that organisms have developed to counter the adverse consequences of oxygen deprivation. The protection it confers against ischemic attack for instance as well as the underlying biological mechanisms have been extensively investigated in animal models. Based on these data, hypoxic conditioning (consisting in recurrent exposure to hypoxia) has been suggested a potential non-pharmacological therapeutic intervention to enhance some physiological functions in individuals in whom acute or chronic pathological events are anticipated or existing. In addition to healthy subjects, some benefits have been reported in patients with cardiovascular and pulmonary diseases as well as in overweight and obese individuals. Hypoxic conditioning consisting in sessions of intermittent exposure to moderate hypoxia repeated over several weeks may induce hematological, vascular, metabolic, and neurological effects. This review addresses the existing evidence regarding the use of hypoxic conditioning as a potential therapeutic modality, and emphasizes on many remaining issues to clarify and future researches to be performed in the field.

## Introduction

Intermittent hypoxia (IH) is well known in clinical physiopathology as a central characteristic of obstructive sleep apnea syndrome (OSAS). Chronic cyclical (30–90 s cycles) severe hypoxia is recognized as a major mechanism underlying the adverse systemic consequences of OSAS. Desaturation–reoxygenation sequences lead to oxidative stress and the production of reactive oxygen species (ROS) (1). Increased ROS levels lead to increased expression of adhesion molecules (2), activation of leukocytes (3), and production of systemic inflammation (4). Oxidative stress, systemic inflammation, and sympathetic activation underlie marked cardiovascular and metabolic morbidities in OSAS. There are now convincing data regarding the association between hypertension, arrhythmias, stroke, coronary heart disease, increased cardiovascular mortality, metabolic dysregulation, and OSAS (5). Nocturnal exposure to IH in healthy subjects has been shown to increase daytime arterial blood pressure and sympathetic activity (6, 7). OSAS should therefore be considered as a systemic disease with IH leading to deleterious consequences throughout the organism.

Several arguments suggest, however, that hypoxic exposure may also lead to some positive adaptations of the human body, able to protect him from several pathological conditions. Athletes have been using hypoxic training as a training strategy for decades, with increasingly complex methods and technology aimed at improving sport performances (8). Interestingly, some epidemiologic data suggest that living at moderate altitude may be associated with lower prevalence of obesity (9–11). Voss et al. (11), for instance, found in a USA-wide representative sample of >400,000 subjects that after controlling for urbanization, temperature category, and behavioral and demographic factors, male and female Americans living <500 m above sea level had 5.1 [95% confidence interval (CI): 2.7–9.5] and 3.9 (95% CI: 1.6–9.3) times the odds of obesity, respectively, compared to their counterparts living >3000 m. Although IH is essentially considered as a deleterious factor in OSAS, some results suggest that moderate OSAS may be associated with some protection against ischemic-reperfusion events (12, 13). IH may in this case enhance the number and function of endothelial progenitor cells, promoting angiogenesis and coronary collateral vessels for instance (14). The potential protective effect of IH exposure also arose from clinical studies, suggesting that patients who had strokes preceded by transient ischemic attacks (implying some hypoxic stress) in the same vascular territory often had less severe deficits at the onset of their stroke and more favorable outcomes (15, 16).

A number of animal studies have described over the past decades the effect of ischemic and hypoxic preconditioning, demonstrating that some pattern and severity of hypoxic exposure can provide protective effects against various deleterious stimuli. While these experimental studies focusing on preconditioning mostly looked at the effect of acute hypoxic conditioning (i.e., a single session of hypoxic exposure) to induce protection over a limited period of time, they opened perspectives regarding the induction of a more prolonged state of protection based on recurrent hypoxic exposure, i.e., hypoxic conditioning (or intermittent hypoxic training). After summarizing the principles of hypoxic (pre)-conditioning, this review will present the available evidence regarding the use of hypoxic conditioning for several pathological conditions as well as highlight the remaining questions and perspectives for future research.

## Principle of Hypoxic (Pre)-Conditioning

Preconditioning is a procedure by which a potentially deleterious stimulus is applied near to but below the threshold of damage to the organism. Shortly after this preconditioning procedure or after some time delay, tissues and organs can develop resistance or tolerance to the same or similar noxious stimuli, therefore preventing or reducing the damage it may induce. The preconditioning phenomenon relies on the basic principle that organisms have developed complex and active defenses to counter adverse conditions such as starvation and oxygen deprivation. Many stimuli, such as ischemia, hypoxia, hypothermia, and pharmacological agents can induce a preconditioning response and modify the responses of the organism to subsequent stress conditions. To identify endogenous mechanisms of protection and repair, and to potentially use these mechanisms therapeutically, preconditioning strategies have been tested over the past decades.

In 1986, Murry et al. (17) first described the principle of ischemic preconditioning. Four cycles of 5-min ischemia followed by short episodes of reperfusion in dogs resulted in a remarkable reduction of infarct size following a subsequently induced myocardial ischemia-reperfusion (I/R) injury. A few years later, Shizukuda et al. (18) were able to demonstrate similar protective effects of hypoxia pre-treatment and ischemic preconditioning and introduced the term hypoxic preconditioning. Both the perfusion of dog hearts with severely hypoxic blood and a short pre-insult period of ischemia of 5 min followed by 10 min of reperfusion subsequently resulted in a comparable reduction of the size of an infarct induced by 60 min of coronary artery occlusion (18).

**Figure 1:**
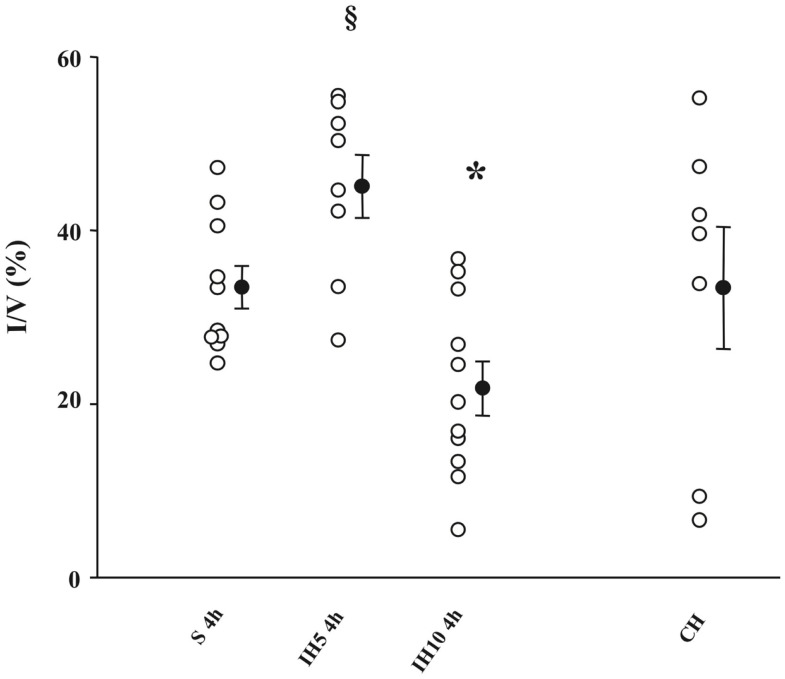
**Infarct size (I) expressed as a percentage of ventricles (V) assessed after a no-flow global ischemia (30 min)-reperfusion (120 min) sequence in groups of mice exposed to 4 h of normoxia (S), intermittent hypoxia (IH, FiO_2_ = 5 or 10%), or chronic hypoxia (CH)**. *P < 0.05 versus the other groups; ^§^*P* < 0.05 versus S; 4 h, and IH10, 4 h. From Ref. (20).

Most of the studies demonstrating IH-induced preconditioning effects in the heart and brain are derived from animal models mimicking OSAS. In a series of studies from our laboratory (19–21), rats were subjected to various paradigms (i.e., pattern and severity of exposure) of acute hypoxia. Compared to normoxia, intermittent moderately severe hypoxia (inspiratory oxygen fraction, FiO_2_ = 10%) applied for 4 h improved the tolerance of the myocardium to ischemia and induced delayed preconditioning by reducing infarct size in isolated rat hearts. Conversely, constant moderately severe hypoxia for 4 h had no protective effect while applying a more severe IH (FiO_2_ = 5%) enhanced infarct size, clearly demonstrating that the effect of IH on infarct size was dependent on the pattern (intermittent versus continuous) and severity (10 versus 5% FiO_2_) of hypoxic exposure (20) (Figure 1). In addition to myocardial protection, similar protective effects of hypoxic preconditioning were also described in the brain, with the discoveries that pre-exposure to hypoxia can prolong anoxic survival by preserving brain metabolism (22), that brain can adapt to anoxia by hypoxic pre-exposure (23), and finally with the description of ischemic preconditioning against ischemic neuronal damage (24). It should be emphasized that most IH exposure used to trigger hypoxic preconditioning induces hypocapnic hypoxemia (because of the hypoxia-induced hyperventilation), while OSAS induces hypercapnic hypoxemia. Hence, the differences between the deleterious consequences of OSAS described earlier and the protective effects of hypoxic preconditioning might result not only from the severity of the IH stress but also from the differences in CO_2_ levels between both conditions.

Most of the studies mentioned above describe a preconditioning phenomenon consisting in acute ischemic or hypoxic exposure (i.e., a single exposure to hypoxia), leading to protection against subsequent ischemic insult for some hours or days only. However, several experimental studies suggest that conditioning strategies using repetitive exposure (several hypoxic exposures over days or weeks) to appropriate doses of hypoxia may induce a prolonged and sustained state of protection.

Hence, as early as 1960, the reported lower incidence of myocardial infarction in people living at high altitude (25) led to animal models describing protective effects of repetitive exposure to IH on the myocardium, more than 10 years (26) before the breakthrough study of Murry et al. on acute ischemic preconditioning (17). The chronic intermittent hypobaric hypoxia model was first designed to reproduce the beneficial effects of adaptation to high altitude. It was characterized by daily exposure to a sustained (4–8 h) period of hypobaric (5000–7000 m) hypoxia followed by a return to normoxic conditions. Intermittence was provided by repetition of this stimulus over several days. Thus, rats exposed to simulated high altitude (5000 m, 8 h/day, 5 days/week, 24–32 days) have reduced I/R-induced myocardial necrosis (27), ventricular arrhythmia (28–30), and apoptosis (31). Overall, chronic intermittent hypobaric hypoxia exposure has consistently been shown to improve cardiac ischemic tolerance, with less adverse effects than chronic continuous hypoxia (32, 33). Most importantly, the myocardial infarct size-limiting effect of chronic intermittent hypobaric hypoxia appears to have a much longer time-span than that obtained with acute preconditioning protocols (34).

Recent studies have been investigating the protective effects of chronic repeated exposure to normobaric hypoxia. Hence, Stowe et al. (35) showed in mice that hypoxic conditioning by repeated hypoxia sequences (2 or 4 h, FiO_2_ = 8 or 11%) for 2 weeks protects against transient focal stroke for the following 8 weeks, together with reduced post-ischemic inflammation. A series of repetitive hypoxic conditioning stimuli (2 h, FiO_2_ = 8 or 11%) for 12 days can induce neuroprotection in the retina that lasts up to 4 weeks (36). Repetitive conditioning stimuli may therefore be an attractive option to provide prolonged protection.

Other results also suggest that intermittent hypoxic exposure may improve regeneration following an organ insult. Hence, conditioning strategies applied after focal ischemia and reperfusion (i.e., post-conditioning) have been proposed as a protective approach to lessen injury and improve recovery. In dogs, following lethal ischemia of the left anterior descending artery, it has been shown that three cycles of 30 s reperfusion and 30 s occlusion at the start of reperfusion reduced myocardial infarct size by 44% (37). Recovery may not only be enhanced by intervention in the minutes following acute ischemia but also by interventions applied over the days following the insult. For instance, in rats after ischemic brain injury IH exposure for 7 days with moderately reduced inspiratory oxygen fraction (FiO_2_ = 12%, 4 h per day) has been shown to enhance neurogenesis and to preserve spatial learning and memory (38). Hence, while acute preconditioning to protect against a subsequent ischemic insult occurring during the following hours has been originally described and most deeply investigated, alternative conditioning strategies include repetitive conditioning stimuli in order to provide prolonged state of protection or regeneration. Such strategies may not only apply to protection or recovery associated with ischemic insults but also to other cardiovascular, metabolic, and neurological disorders (see below). Hypoxic exposure can be used to trigger conditioning phenomenon when applied at rest but also when combined with exercise (exercise training in hypoxia, see below) or with hyperoxic exposure [intermittent hypoxic–hyperoxic training, e.g., Ref. (39, 40)].

## Potential Mechanisms Triggered by Hypoxic Conditioning

As mentioned above, the time-course of protection afforded by hypoxic conditioning can be extended by increasing the duration of the conditioning protocol (34). This could be due to the recruitment of additional protective mechanisms in response to repetitive exposure. In this section, we review the various mechanisms involved in the beneficial effects of both acute and long-term IH exposure in order to propose a potential explanation for this phenomenon.

Whether acute or repeated, hypoxic conditioning appears to be accompanied by substantial changes in gene expression. A crucial mediator of this genomic response is the hypoxia-inducible factor 1 (HIF-1) transcription factor, a key regulator of cellular oxygen homeostasis. HIF-1 is a complex protein composed of two subunits, the cytosolic HIF-1α (O_2_-sensitive) subunit and the nuclear HIF-1β subunit, which dimerize in the nucleus to form the functional HIF-1 transcription factor and activate gene transcription. In well-oxygenated cells, proline hydroxylation of the HIF-1α subunit by prolyl-hydroxylase enzymes (PHDs) leads to ubiquitination and proteasomal degradation. In sustained hypoxic conditions, HIF-1α hydroxylation and subsequent degradation are reduced because of substrate (O_2_) limitation, so that HIF-1α translocates to the nucleus to bind HIF-1β and activate the transcription of various genes geared to maintain cellular homeostasis under reduced oxygen availability. Upon return to normoxic conditions, cytosolic levels of HIF-1α rapidly decline and the effects of HIF-1 decrease (41).

The major role of HIF-1 and its target genes in ischemic/hypoxic conditioning is evidenced by the complete loss of ischemia-induced cardioprotection in mice with partial HIF-1 deficiency (42). Similarly, we have shown that prevention of HIF-1 activation abolished the delayed cardio-protection provided by acute IH in the rat (21), and a recent study reported that HIF-1 inhibition prevented the acute IH-induced neuroprotection (43).

Nitric oxide (NO) is known to play a critical role in preconditioning and cytoprotection through its vasodilating effect as well as for its ability to modulate mitochondrial function (44). Others and we have shown that the inducible nitric oxide synthase (iNOS) gene, a HIF-1 target gene, is up-regulated along with HIF-1 activation in response to acute exposure to IH (21, 45) and that NO is involved in its delayed protective effects (20, 21, 46). In addition to HIF-1, GATA-4, a transcription factor involved in the regulation of apoptosis, has been shown to activate the gene expression of the anti-apoptotic proteins, bcl-2 and bcl-x(L), following acute IH exposure in mice (47). Finally, well-known mediators of classic ischemic preconditioning are also triggered by acute IH exposure. Hence, we have evidenced the role of ATP-dependent potassium (K_ATP_) channels (20) and of stress-activated kinases such as protein kinase C (PKC), p38 mitogen-activated protein kinase (MAPK), and extracellular signal-regulated kinase (ERK1/2) (19) in the cardio-protection induced by a 4-h exposure to moderately severe (FiO2 = 10%) IH.

Although the effects of HIF-1 rapidly decline upon re-oxygenation following sustained hypoxia, we have found that IH induces a strong and sustained whole-body activation of HIF-1 that persists after IH exposure. This can be explained by the oxidative stress produced by the repetition of oxygenation-desaturation sequences. Indeed, ROS are potent inductors of HIF-1 by increasing the expression of the HIF-1α protein and by inhibiting its PHD-dependent degradation (41). Repeated IH exposure could thus result in activation of additional cytoprotective genes that are not or insufficiently expressed upon sustained hypoxia or short-term IH exposure.

Consequently, in addition to involving the iNOS gene (48) and the K_ATP_ channels (49), chronic IH has also been shown to promote the expression of the erythropoietin (EPO) gene (50). EPO is well recognized as a potent protective agent against ischemic injury since its binding to EPO receptors activates numerous protective signaling pathways, such as Janus kinase 2 (JAK2)/signal transducer and activator of transcription 3 (STAT3) pathway, the phosphatidylinositol 3-kinase (PI3K)/protein kinase B (Akt) pathway, and the MAPK pathway, all known to confer cytoprotection, in particular cardioprotection (51), nephroprotection (52), and neuroprotection (53). A recent study confirms the pivotal role of the JAK2/STAT3 pathway in a cardioprotective chronic IH model. Activation of the pathway by a 4-week exposure to chronic IH was directly linked to improvement of functional recovery upon reperfusion via maintenance of intracellular Ca^2+^ homeostasis and of mitochondrial function (54). The involvement of other EPO-related signaling pathways, such as the PI3K/Akt pathway, has also been confirmed in the infarct size-sparing effects of chronic IH exposure (55, 56). Interestingly, we observed that (PI3K)/Akt pathway was not involved in the cardioprotection afforded by acute IH exposure (19). This is in agreement with the notion that long-term exposure can trigger additional protective mechanisms, particularly through EPO production. In accordance, EPO has been shown to play a pivotal role in the cerebral infarct size reduction provided by chronic IH post-conditioning in mice (57).

Among the other tissue protective genes upregulated by HIF-1 upon long-term hypoxic exposure are genes encoding angiogenic growth factors and cytokines, such as vascular endothelial growth factor (VEGF), angiopoietins, and platelet-derived growth factors (41). In agreement, rats exposed to chronic IH display a 1.5-fold increase in left ventricular capillary, a higher pre-ischemic coronary flow of isolated hearts and an improved recovery after I/R (58). We also found an increased myocardial capillary density and VEGF expression in rats exposed to 35 days of normobaric IH (59). This increase in angiogenesis might not only contribute to the cytoprotection conferred by repeated hypoxic conditioning but could also explain why the protection lasts longer than that obtained with acute conditioning protocols.

Finally, other important mediators of the cytoprotective effects of chronic IH are heat stress proteins (HSP), in particular the HSP70 family, also regulated by HIF-1 (60). HSP70 are thought to act as molecular chaperones to repair or remove proteins denatured by stresses such as I/R, leading to protection and/or restoration of cell function (61). Thus, hypoxic preconditioning has been shown to reinforce HIF-1-dependent HSP70 signaling with beneficial effects on ischemic renal apoptosis and autophagy (60) as well as occurrence of I/R-induced ventricular arrhythmias (62).

## Hypoxic Conditioning in Healthy Subjects

Intermittent hypoxia training is recognized by the sports medicine community as a potentially useful strategy to enhance exercise performance in athletes (8). Living or training under hypoxic conditions may improve exercise performance probably by promoting hematological and muscle adaptations and without eliciting the detrimental effects of chronic hypoxic exposure. Several studies focusing on these types of hypoxic training programs reported promising results especially in terms of performance gain, but some debates remain regarding the usefulness of IH training in athletes (63). Beyond the enhancement of physical performances (8, 64), and on the basis of some established protective preclinical effects of IH, hypoxic conditioning in healthy subjects has been evaluated as a potential useful intervention to improve some physiological functions and risk factors for acute and chronic diseases.

### Hematology

As demonstrated following environmental exposure to sustained hypobaric hypoxia (e.g., altitude) (65), moderate IH exposure can also trigger hematological changes. Nine days of hypobaric IH exposure (simulated altitude ranging from 4000 to 5500 m, 3–5 h/day) induced a significant increase in hematocrit, red blood cell count, reticulocytes, and hemoglobin concentration (66). Hellemans (67) showed that normobaric IH sessions (5 min FiO_2_ = 9–10% alternating with 5 min of normoxia, 60 min per day, twice a day, for 18 days) in 10 elite endurance athletes triggered significant increases in reticulocyte count (30%), hemoglobin (4%), and hematocrit (5%). Hence, while continuous and prolonged exposure to hypoxia triggers significant and sustained hematological effects, IH protocols with substantially lower total hypoxic doses seem to be sufficient to enhance erythropoiesis (68).

### Ventilation

Beyond the well-known increase in hypoxic ventilatory response associated with prolonged hypoxic exposure, IH may elicit specific forms of respiratory plasticity. Briefly, respiratory plasticity is defined as a persistent change in the neural pathways and synapses (morphology and/or function) of the nervous system involved in generating respiratory activity, in response to prior experience (69). In the context of IH, respiratory plasticity is referred to long-term facilitation (LTF) (70). Ten brief bouts (3 min) of isocapnic hypoxia (FiO_2_ = 8%), interspersed with 5 min of normoxia applied during non-rapid eye movement sleep in healthy subjects (71) and snoring individuals (72) reduced upper airway resistance, suggesting LTF of upper airway dilatators and more particularly hypoglossal muscle LTF (73). Hence, based on these results suggesting that IH exposure may trigger respiratory plasticity in physiological conditions, translating the use of hypoxic conditioning to pathological conditions seems to be relevant and will be discussed later in this review.

### Cardiovascular system

In contrast to the harmful effects of chronic IH upon the cardiovascular system observed in severe OSAS, moderate IH may induce some positive cardiovascular adaptations. Shatilo et al. (74) submitted two groups of healthy 60- to 74-year-old men (14 physically active, 21 sedentary) to normobaric IH (cycles of 5 min hypoxia, FiO_2_ = 12%, followed by 5 min normoxia, repeated four times a day during 10 days). In sedentary subjects only, IH induced a decrease in blood pressure of 7.9 ± 3.1 mmHg and an increase in submaximal exercise capacity (workload at anaerobic threshold + 12.7%), highlighting the positive cardiovascular effects of hypoxic conditioning in healthy older inactive men. Supporting these results, and by combining hypoxic exposure and exercise, Bailey et al. (75) exposed, in a randomized controlled and double blind manner, 34 physically active subjects to either a normoxic (*n* = 14, FiO_2_ ≈ 20.9%) or a hypoxic (*n* = 18, FiO_2_ ≈ 16%) training (cycling three times per week for 20–30 min at 70–85% of maximum heart rate previously determined either in normoxia or hypoxia, during 4 weeks). Hypoxic exercise training only significantly decreased maximal exercise systolic blood pressure by 10 ± 9 mmHg and increased maximal oxygen uptake by 0.47 ± 0.77 L/min.

Increased arterial stiffness is known to be associated with an increase in the risk of occurrence of cardiovascular events (76). The beneficial aspects of combining hypoxic exposure and exercise training upon arterial stiffness have been studied in women. Sixteen postmenopausal women were randomly allocated either to a hypoxic exercise group (*n* = 8, hypobaric hypoxic chamber, 2000 m, 2 h exposure per session, 4 days per week, during 8 weeks) or a normoxic exercise group (*n* = 8, 2 h exposure per session, 4 days per week, during 8 weeks). Exercise (aquatic exercises) was performed in both groups under normoxic or hypoxic conditions at an intensity of ≈50% peak oxygen uptake for 30 min. Mild hypoxic exposure combined with exercise training significantly reduced arterial stiffness in postmenopausal women, while exercise training performed at the same relative intensity under normoxic conditions did not induce any change (77). These results corroborate the work of Vedam et al. (78) showing an NO-mediated reduction in arterial stiffness among healthy adults men exposed to a single hypoxic session (*n* = 12, arterial oxyhemoglobin saturation ≈ 80% during 20 min) compared to subjects evaluated under room-air conditions.

Taken together, these results confirm the potential positive effects of hypoxic conditioning on the cardiovascular function in healthy subjects, including older individuals and females.

### Metabolic status

Preclinical data indicate a potential beneficial impact of moderate IH exposure on blood glucose and cholesterol levels, mitochondrial enzyme activity, glycolysis, and fatty-acid oxidation (79–81). In healthy humans also, some data suggest that IH either at rest or combined with exercise training can improve metabolic status. In a single blind, randomized controlled trial, Haufe at al. (82) have shown in 20 healthy lean subjects that performing endurance training in hypoxia (60 min of treadmill running, three times a week during 4 weeks, FiO_2_ = 15%) compared to normoxia (FiO_2_ = 21%) at the same relative intensity elicit greater reduction in body fat mass as well as in circulating triglyceride and fasting insulin levels. Interestingly, these effects were observed despite of a smaller absolute workload during hypoxic exercise training.

Intermittent hypoxia exposure can also significantly modify appetite regulation. Bailey et al. (83) reported that a 3 h exposure to normobaric hypoxia while performing a 50 min exercise in 12 healthy males caused a suppression in appetite and a reduction in plasma levels of acylated ghrelin concentrations, the hunger-stimulating hormone.

## Hypoxic Conditioning for Patients

Based on preclinical data as well as on studies in healthy subjects showing potential positive effects of IH exposure, pilot investigations have been conducted in various pathological conditions to assess the potential clinical application of hypoxic conditioning (Table 1).

**Table 1:** **Characteristics of hypoxic conditioning interventions in patients**.

Author	Subjects	Conditioning stimulus			
		Duration	Hypoxic intervention	Control intervention	Exercise/rest
**CARDIOVASCULAR DISEASES**					
Burtscher et al. (84)	16 elderly men (50–70 years old, eight with prior myocardial infarction, and eight without)	3 weeks, 5 sessions a week (15 sessions)	Three to five times 3-5 min hypoxia (FiO_2_ = 10–14%) – 3 min normoxia	Normoxia (3 weeks of exposure, five sessions a week)	Rest
Lyamina et al. (85)	37 young non-overweight men with stage 1 hypertension (32 years old)	20 consecutive days, one session a day	Four to 10 times 3 min hypoxia (FiO_2_ = 10%) – 3 min normoxia	20 normotensive participants (35 years old), no exposure	Rest
**NEUROLOGICAL DISEASES**					
Trumbower et al. (86)	13 subjects with incomplete SCI, ASIA Score C or D	One single session	15 times 60 or 90 s hypoxia (FiO_2_ = 9%) – 60 s normoxia	Normoxia	Rest
Schega et al. (87)	34 healthy subjects (60-70 years), cognitively preserved	6 weeks, 3 sessions a week (18 sessions)	1 h/session, 10 min hypoxia (FiO_2_ adjusted to reach a SpO_2_ = 90% the first 2 weeks, 85% the third week and 80% the last three weeks) – 5 min normoxia	Normoxia	30 min strength-endurance training after each normoxia/hypoxia session
Hayes et al. (88)	19 subjects with incomplete chronic SCI, ASIA Score C or D	5 consecutive days, one session a day	15 times 90 s hypoxia (FiO_2_ = 9%) – 60 s normoxia	Normoxia	Conditioning at rest or combined with a 30-min over-ground walking training performed 1 h later
Tester et al. (89)	Eight individuals with cervical or thoracic incomplete SCI, ASIA Score ranging from A to D	10 consecutive days, one session a day	Eight times 2 min hypoxia (FiO_2_ = 8%) – 2 min normoxia, controlled end-tidal CO2 level (2 mmHg above resting values)	Normoxia in a subset of *n* = 4 subjects 1 day before and 10 days after conditioning	Rest
**VENTILATION/RESPIRATORY DISEASES**					
Aboubakr et al. (90)	11 severe OSAS patients	One night session during non-rapid-eye movement sleep before and after 4 weeks of CPAP treatment	10 times 3 min hypoxia (FiO_2_ = 8%) – 5 min normoxia, isocapnia	One-night normoxia, subset of *n* = 7	Rest
Rowley et al. (91)	13 OSAS patients	One night session during non-rapid-eye movement sleep	10 times 3 min hypoxia (FiO_2_ = 8%) – 5 min normoxia, isocapnia	One-night normoxia, subset of *n* = 8	Rest
Burtscher et al. (92)	18 eucapnic normoxic mild COPD patients	3 weeks, 5 sessions a week (15 sessions)	3 to 5 times 3-5 min hypoxia (FiO_2_ = 12-15%) – 3 min normoxia	Normoxia	Rest
Haider et al. (93)	18 eucapnic normoxic mild COPD patients	3 weeks, 5 sessions a week (15 sessions)	Three to five times 3–5 min hypoxia (FiO_2_ = 12–15%) – 3 min normoxia	Normoxia Age-matched healthy controls, *n* = 14	Rest
**METABOLIC DISEASES**					
Netzer et al. (94)	20 obese subjects	8 weeks, 3 sessions a week (24 sessions)	90 min hypoxia (FiO_2_ = 15%)	Normoxia, sham-exposure	Stepper, treadmill and bicycle ergometer-training combined with conditioning, without any dietary intervention
Wiesner et al. (95)	45 sedentary overweight or obese subjects, non-diabetic or insulin-resistant	4 weeks, 3 sessions a week (12 sessions)	60 min hypoxia (FiO_2_ = 15%)	Normoxia	Treadmill-training combined with conditioning
Mackenzie et al. (96)	Eight type 2 diabetic patients	One single session	60 min hypoxia (FiO_2_ = 15%)	Normoxia	Four conditions for all patients: Rest: hypoxia and normoxia; Cycling: hypoxia and normoxia
Mackenzie et al. (97)	Eight type 2 diabetic patients	One single 60, 40 and 20-min session, combined with exercise	60, 40 and 20 min hypoxia (FiO_2_ = 15%)	No control group	60, 40, and 20 min cycling
Workman and Basset et al. (98)	15 sedentary overweight males	One session. A subset of *n* = 6 underwent conditioning on 6 consecutive days	3 h hypoxia, targeted SpO_2_ ≈ 80%	3 h normoxia	Rest
Kong et al. (99)	18 young obese subjects	4 weeks, 3 sessions a week, cumulative exposure of 6 h/week	2 h hypoxia (FiO_2_ = 14.5-16.4%)	2 h normoxia	Endurance and strength training combined with a low-calorie intake diet

*ASIA, American Spinal Injury Association; COPD, chronic obstructive pulmonary disease; FiO_2_, fraction of inspired oxygen; h, hour(s); min, minute(s); Normoxia (FiO_2_ = 21%); OSAS, obstructive sleep apnea syndrome; s, second(s); SCI, spinal cord injury; SpO_2_, peripheral capillary oxygen saturation*.

### Cardiovascular diseases

#### Hypertension and Vascular Function

Murine models have demonstrated the antihypertensive effect of hypoxic conditioning as observed in spontaneously hypertensive rats. This effect seems to be mediated, at least in part, by NO metabolism, which may underlie hypoxia-induced prevention of endothelial dysfunction, a well-known major risk for hypertension (100–102). IH has been proposed as a mean to treat hypertension in humans. Serebrovskaya et al. (102) highlight in their review encouraging results reported in the Russian literature with noteworthy improvements in blood pressure (e.g., a decrease of 10–30 mmHg in systolic and 10–15 mmHg in diastolic blood pressure) following exposure to only a few episodes of moderate hypoxia (FiO_2_ = 10–14%) for a short duration (15 min to 4 h per session) for 10–30 days. In a more recent study, Lyamina et al. (85) exposed 37 young non-overweight men with stage 1 hypertension to 20 sessions of 4 to 10 cycles of 3-min hypoxia (FiO_2_ = 10%) and 3-min room air breathing. A significant decrease in blood pressure was observed following the intervention with values in patients not different anymore from the normotensive control group. Moreover, the reduction in blood pressure persisted for up to 3 months in 28 initially hypertensive patients. Thus, considering the good tolerance profile and apparently harmless aspect of moderate IH conditioning protocols, it could be considered as an interesting and promising adjunct therapeutic strategy to reduce systemic blood pressure.

#### Acute Myocardial Infarction

In addition to its potential effect on the vascular function and blood pressure, hypoxic conditioning strategies may be useful to improve heart function and prevent the early and delayed deleterious consequences of myocardial infarction. Forty days of hypobaric IH exposure before ligation of the left coronary artery in mice reduced myocardial infarct size and mortality rates (26). The same intervention applied after ligation was also shown to reduce the risk of arrhythmia occurrence (29). Normobaric IH protocols induce the same effects, with reduced cardiac arrhythmias during ischemia and decreased infarct size by 43% following ischemia-reperfusion injury (103). However, in humans, since myocardial infarction is not a predictable event, similar hypoxic preconditioning strategies using IH are actually difficult to implement.

Besides hypoxic conditioning, ischemic conditioning, consisting in brief ischemic episodes, even remote (lower or upper limb intermittent ischemia), can enhance myocardial tolerance to subsequent ischemic or ischemia-reperfusion insults. Based on preclinical remote or perioperative ischemic conditioning studies (17, 104), clinical studies have been conducted to evaluate the effect of remote ischemic conditioning (RIC) in patients. In a randomized controlled study, 333 consecutive adult patients with a suspected first acute myocardial infarction were assigned to receive a primary percutaneous coronary intervention with (interventional group, unilateral intermittent upper-arm ischemia, four cycles of 5 min inflation up to 200 mmHg and 5 min deflation of a standard upper-arm pressure cuff) or without (control group) prior RIC during transport to hospital. In addition to the favorable safety profile of RIC, a significant increase in myocardial salvage, an established prognostic factor of mortality (105), has been shown when RIC was applied (106). More recently, and using the same study design and RIC paradigm in patients presenting with a ST-segment elevation myocardial infarction, the group treated with RIC prior to primary percutaneous coronary intervention showed reduced myocardial infarct size, increased myocardial salvage, and reduced myocardial edema (107).

#### Chronic Coronary Artery Disease

The beneficial role of IH in chronic coronary artery disease has also been considered (32). An augmented coronary collateral vessel development has been reported in OSAS patients (apnea-hypopnea index >10 events/h) (13) suggesting that some degree of IH may induce positive myocardial adaptations. In a prospective controlled study in 16 elderly men (50–70 years old, eight with prior myocardial infarction and eight without), subjects were assigned randomly and in a double-blind fashion to moderate IH [3 weeks, 15 passive exposure sessions, three to five episodes (3–5 min) of hypoxia (FiO_2_ = 10–14%) per session with 3-min normoxic interval] or normoxia (control group, 3 weeks of exposure). An increased peak-oxygen consumption following hypoxic conditioning was shown both in subjects with and without prior myocardial infarction compared to normoxic exposure (84). Furthermore, heart rate, systolic blood pressure, blood lactate concentration, and the rating of perceived exertion during submaximal exercise (cycling at 1 W/kg) were diminished in subjects exposed to IH. Hence, IH exposure may be considered in chronic coronary artery disease as a procedure to improve cardiovascular parameters.

#### Heart Failure

Preclinical studies in healthy lean mice (108) and in a mice model of heart failure (109) showed an improved cardiac function following 4 weeks of exposure to IH. In patients with heart failure, a syndrome resulting from several cardiac injuries, IH has not been clinically tested yet, but encouraging results regarding RIC are actually available. Kono et al. (110) reported increased coronary flow reserve both in subjects with heart failure (*n* = 10) and in healthy subjects (*n* = 10) following 1 week of upper-limb RIC (4 cycles of 5 min inflation and 5 min deflation of a blood pressure cuff).

While current preclinical and clinical results suggest that IH may be an innovative and non-pharmacological therapeutic option in cardiovascular diseases, additional clinical studies are required in patients with various cardiac conditions to confirm the potential of hypoxic conditioning as a therapeutic tool.

### Neurological diseases

#### Stroke

The protective effects of hypoxic conditioning are not restricted to the heart and may also apply to the brain. Remarkable clinical and preclinical data support the ability of hypoxic/ischemic conditioning to enhance brain tolerance to ischemia. In humans, retrospective studies (15, 16) or prospective studies (111) and clinical evidence (112, 113) have reported lower severity and better functional outcome following stroke in patients who have previously experienced spontaneous transient ischemic attacks. Despite some discrepancies (114–116), these findings suggest an endogenous ischemic neuroprotective preconditioning triggered by transient ischemic attacks. As for myocardial infarction, the unpredictable nature of stroke actually restrains the clinical applicability of hypoxic/ischemic preconditioning. Post-conditioning might be a useful alternative paradigm. It refers to the application of the conditioning stimulus after the occurrence of the harmful event in order to treat or prevent its consequences. In rodents, 7 days after transient mild cerebral artery occlusion, exposure to moderate IH for 7 days induced hippocampal neurogenesis, synaptogenesis, increased brain-derived neurotrophic factor expression, and significantly improved functional outcomes regarding spatial learning and memory (117). RIC following acute ischemic stroke has also been studied in preclinical (118) and clinical (119) conditions alone or as an adjunct therapy to conventional fibrinolysis with encouraging results in both cases. Since hypoxic/ischemic conditioning seems to be harmless and relatively easy to implement at bedside, it may represent a promising adjunct therapy in stroke patients.

#### Spinal Cord Injury

Trumbower et al. (86) exposed, in a randomized controlled single blind crossover trial, 13 incomplete spinal cord injury (SCI) subjects to 15 repeated bouts of IH (60 or 90 s, FiO_2_ = 9%), separated by 60 s of normoxic exposure (FiO_2_ = 21%). Hypoxic sessions were compared to sessions in which subjects received sham exposure (i.e., room air). Changes in maximum isometric ankle plantar flexor torque generation were significantly increased by 82 ± 33% immediately after IH exposure and were maintained above baseline for more than 90 min. Increased ankle plantar flexor electromyogram activity was correlated with increased torque (*r*^2^ = 0.5, *P* < 0.001). There was no change from baseline following sham experiments. Hayes et al. (88), in a randomized, double blind, placebo-controlled, crossover study, exposed 19 participants with chronic incomplete SCI to 15 repetitions of 90 s hypoxic exposure (FiO_2_ = 9%) interspersed by 60 s normoxia or to normoxic exposure (FiO_2_ = 21%, placebo) on five consecutive days, alone or combined with 30 min of over-ground walking 1 h later. In this study, IH improved both walking speed and endurance, and the impact of hypoxia exposure was enhanced when combined with walking (88). Beyond motor function enhancement, IH could also promote recovery of respiratory motor function: a recent case study has outlined an improvement in airflow in response to resistive loads applied in a 55-year-old woman with chronic SCI following acute exposure to IH (120). Furthermore, in a controlled trial, eight individuals with either cervical (*n* = 6) or thoracic (*n* = 2) incomplete SCI were exposed to IH [eight 2 min hypoxic bouts (FiO_2_ = 8%) interspersed with a 2 min recovery period (room air, FiO_2_ = 21%)] for 10 consecutive days, with controlled end-tidal CO_2_ level (2 mmHg above resting values) (89). A subset of four participants received additional sham exposure (room air). After each single IH session, an increase in minute ventilation 30 min after IH exposure was observed, whereas no change was observed following sham exposure to room air. These results occurred within the first 2 days of IH exposure, and persisted throughout the 10 days of exposure to IH. The magnitude of ventilatory LTF remained enhanced 10 days after the intervention in some (*n* = 2) but not all participants.

The effect of hypoxic conditioning in the field of cognitive performances has also been explored in healthy older individuals. In a recent randomized controlled trial, 34 retired healthy subjects (60–70 years), not physically active and cognitively preserved (MMSE > 27/30), were assigned either to a normoxic group (control group, *n* = 17, targeted SpO_2_ = 94–98%) or to a hypoxic group (*n* = 17, alternating 10 min hypoxia and 5 min of normoxia, FiO_2_ in hypoxia was adjusted to reach a SpO_2_ = 90% the first 2 weeks, 85% the third week, and 80% in the last 3 weeks during hypoxia). Both groups were subjected to 1-h sessions (normoxia/hypoxia), three times a week for 6 weeks. Thirty minutes strength-endurance training followed each normoxic/hypoxic session. Combining IH exposure and exercise training enhanced cognitive performance and quality of life to a greater extent than exercise training alone (87).

On the basis of these clinical findings and according for instance to promising results in animal models of Alzheimer’s disease submitted to IH (121), further research is needed to evaluate the effect of IH exposure in neurological conditions and the potential of hypoxic conditioning as a preventive or therapeutic tool for these diseases. As physical activity, by triggering beneficial neurovascular adaptations (122), hypoxic conditioning may be a promising therapeutic strategy to prevent or slow down brain aging.

### Respiratory diseases

Although exposing patients with respiratory illnesses to IH may seem provocative, some data suggest that hypoxic conditioning may also be considered for these patients.

#### Obstructive Sleep Apnea Syndrome

Considering OSAS patients, a decrease in upper airway resistance following moderate IH protocol [11 subjects with severe OSAS, exposed during non-rapid eye movement to 10 cycles of 3-min hypoxia (FiO_2_ = 8%) followed by 5 min of room air] has been shown, indicating a LTF of upper airway dilatators (90). The same IH paradigm does not alter upper airway critical closing pressure within the same population (91). This may support the use of IH as an adjunct therapy in OSAS, in combination with other conventional treatments (e.g., continuous positive airway pressure), as suggested by Mateika et al. in their recent review (123). Additional work is, however, required to optimize IH protocols before translating it to therapeutic interventions.

#### Chronic Obstructive Pulmonary Disease

The effects of repetitive bouts of mild acute IH [15 sessions over 3 weeks, three to five hypoxic bouts (FiO_2_ = 12–15%), each lasting 3–5 min, separated by 3 min normoxic intervals], were evaluated in 18 eucapnic normoxic mild chronic obstructive pulmonary disease (COPD) patients according to a randomized controlled and double blind study design. Increased total hemoglobin mass, exercise time to anaerobic threshold, and total exercise time were demonstrated (92) as well as improved baroreflex sensitivity up to normal levels and enhanced hypercapnic ventilatory response without changes in hypoxic ventilatory response (93).

### Metabolic diseases

The use of IH to induce weight loss has been suggested since hypoxia can increase energy expenditure (98, 124). Obese subjects submitted to hypobaric hypoxia (1 week at 2650 m) showed weight loss, increased basal metabolic rate, and decreased food intake (125). Combining hypoxic exposure with physical activity has been reported to potentiate weight loss. Netzer et al. (94) randomly exposed 20 obese individuals (mean body mass index = 33.1 kg/m^2^) to an 8-week training program (three 90 min sessions per week) at 60% of their individual maximal oxygen uptake either in normobaric hypoxia (FiO_2_ = 15%) or in normoxia (sham, FiO_2_ = 20.1%), without any dietary intervention. Subjects in the hypoxic group lost significantly more weight (1.14 versus 0.03 Kg) than the sham group. Wiesner et al. (95) submitted sedentary, non-diabetic or insulin-resistant, overweight or obese subjects to a treadmill-training, 60 min a day, thrice weekly over a 4-week period at a heart rate corresponding to 65% of their maximum oxygen uptake under normoxia (normoxic group, *n* = 21, FiO_2_ = 21%) or normobaric hypoxia (hypoxic group, *n* = 24, FiO_2_ = 15%). Both groups showed similar improvement in maximal oxygen uptake, despite a smaller training workload in the hypoxic group. Body composition improved more (i.e., greater reduction in body-fat mass) in the hypoxic group. More recently, Kong et al. (99) randomly assigned 22 young obese subjects (17–25 years, body mass index >27.5 kg/m^2^) to either a normobaric hypoxic (FiO_2_ = 16.4–14.5%, three 2 h sessions per week, cumulative exposure of 6 h/week) or a normoxic (FiO_2_ = 21%) training for 4 weeks. Combined with a low-calorie intake diet and a strength-endurance training program, both groups exhibited weight loss, but weight loss was significantly greater in the hypoxic group. In addition, hypoxic exposure improved systolic blood pressure and mean blood pressure. Taken together, these arguments are supportive of IH as an adjunct therapy to enhance weight loss in obese patients. In a recent review, Urdampilleta et al. (126) proposed an IH conditioning protocol targeting both weight loss and aerobic capacity improvement. This protocol would consist in IH exposure three times per week, during 3–6 weeks, with a target FiO_2_ of 10–12%, with or without physical training (20–30 min strength-resistance exercises and 30 min high-intensity aerobic exercise).

Based on preclinical data (127), hypoxic conditioning has been tested in type 2 diabetes. Eight type 2 diabetic patients completed four 60-min sessions (interspersed by 7–14 days) of normoxic rest, normoxic exercise, hypoxic rest, and hypoxic exercise (FiO_2_ = 14.6 ± 0.4%) (41). Exercise intensity was set at 90% of lactate threshold. Acute hypoxic exposure both at rest and during exercise increased insulin sensitivity (96). In another study from the same group, eight type 2 diabetic males first performed 60 min of hypoxic exercise (FiO_2_ = 14.7 ± 0.2%, exercise intensity at 90% of lactate threshold = 49 ± 4 Watts) and then 40- and 20-min exercise sessions at a higher intensity (at 70 ± 9 and 140 ± 12 Watts, respectively) to provide similar total workload. This protocol induced an acute- (24 h) and moderate-term (48 h) improvement in insulin sensitivity following the 40- and 60-min hypoxic exercise sessions. This emphasizes the role of exercise duration or intensity (rather than the total workload completed) on glucose control in type 2 diabetics (97). Although encouraging, the beneficial effects of hypoxic conditioning for diabetic patients have nonetheless to be further evaluated by additional clinical studies.

## Remaining Questions and Perspectives for Future Researches

**Figure 2:**
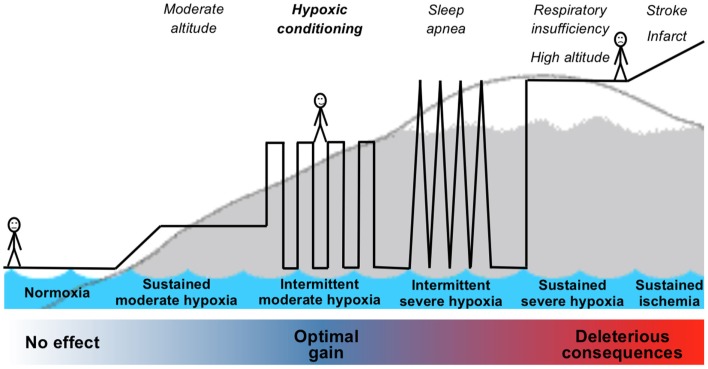
**Schematic representation of the continuum from normoxia to severe hypoxia including hypoxic exposure leading to hypoxic conditioning**.

Although the bench-based foundation for ischemic and hypoxic conditioning is strong, the translation of conditioning strategies into effective, validated therapies and clinical practice is so far mostly missing. The above results reported in patients, despite frequent important weakness in study design, suggest several promising applications for hypoxic conditioning as a non-pharmacological intervention for prevention or recovery in many pathological conditions. To successfully address the challenge of translating the strong experimental and preclinical data regarding conditioning strategies into potential clinical applications, several important issues have to be considered. Many questions remain regarding the optimal conditioning stimulus, the most favorable clinical setting to apply the conditioning phenomenon, and whether a conditioning response can always be induced in humans who, in contrast to laboratory animals, are elderly and have multiple comorbidities. Since hypoxic conditioning is part of a continuum from normoxia to deleterious severe hypoxia (Figure 2), suitable biomarkers to identify in humans the occurrence of hypoxic conditioning are needed.

Various conditioning stimuli such as exposure to ischemia, hypoxia, metabolic inhibition, or inflammation below the threshold of damage have been tested mostly in animal models and in some cases in humans. Several conditioning strategies involved drug administration and were potentially associated with important limitations in terms of clinical application due to tolerance issues, potential adverse effects, and clinical approval. Conversely, non-pharmacological ischemic or hypoxic conditioning strategies provide reasonable safety/toxicity profiles and appear to be well tolerated in patients. Among the non-pharmacological conditioning strategies, RIC (e.g., limb ischemia to induce organ protection) has been mostly tested in recent large clinical studies. Although hypoxic conditioning and RIC may induce some common mechanisms, these two attractive and relatively easy to implement conditioning strategies have to be compared in order to determine which one should be the most efficient and practical for the clinic.

Although preclinical studies demonstrated the striking ability of preconditioning strategies to protect against ischemic injuries, in most clinical settings demonstration of the therapeutic potential of preconditioning against major insults may not be readily achievable. Stroke as well as cardiac arrest is mostly of unpredictable nature, which makes clinical validation of anticipatory treatment with preconditioning strategy difficult. In addition, most preconditioning models induce short-term protection (i.e., <72 h), while more prolonged state of protection or regeneration are needed for many clinical settings. Hence, clinical validation and application of new conditioning strategies inducing sustained state of protection and regeneration such as prolonged hypoxic conditioning may be most appropriate (i) to improve risk factors for cardiovascular, metabolic, and neurological diseases in populations not yet presenting acute insults or advanced degenerative diseases (e.g., older individuals), (ii) as an intervention aiming to antagonize and even reverse degenerative processes associated with chronic disorders (e.g., neurological disorders), and (iii) as a regenerative treatment following acute ischemic insult (e.g., following infarct).

The dose response of a conditioning stimuli is thought to range from no response at low intensities to a protected state at higher intensities while a further increase in stimulus intensity would cause tissue damage. The therapeutic range of conditioning may be therefore relatively narrow. Although grouped within the term hypoxic conditioning, the interventions used in the human studies described above vary greatly in terms of hypoxic exposure duration, frequency, and severity (Table 1). Since all stimuli are defined by their frequency, magnitude, and duration, optimal titration of the repetitive stimulus is critical in order to provide proofs of concept and practical guidelines for hypoxic conditioning.

Responses to conditioning stimuli might be specific to sex, dependent on age, and affected by medical comorbidities. Large inter-individual differences exist in the response to identical hypoxic stimulus due to variations in oxygen regulated gene expression. This was shown for VEGF and other downstream genes of HIF-1, suggesting that the source of this variation resides within the HIF system itself (128). There are also epigenetic differences between individuals as well as environmental and life style related variables that may affect the hypoxic conditioning mechanisms. Titration to define the optimal individual dose of hypoxia for subsequent hypoxic conditioning program may be needed to obtain the largest benefits without adverse effects.

Appropriate titration of the conditioning stimulus will depend on reliable and validated biomarkers that remain to be determined. It is therefore critical to identify suitable biomarkers and most probably arrays of biomarkers to detect whether a conditioning response is actually occurring or conversely whether some deleterious consequences of IH are induced as well as to distinguish responders and non-responders to a given conditioning strategy. Further animal studies are needed to determine such biomarkers specific to hypoxic conditioning, which should subsequently be validated in humans.

There is an increasing number of commercially available devices permitting hypoxic exposure. These devices use technologies to reduce inhaled oxygen concentrations from the normal 21% to below 10%. Exposure can be done by hypoxic breathing through a mask or inside a confined space like tents or rooms while resting, sleeping, or exercising. These products are however mostly designed for athletes and healthy individuals. The use of hypoxic conditioning as a therapeutic strategy in patients would require the use of specific technologies permitting controlled, safe, and inexpensive IH exposure. Although the technologies mostly exist, their integration within validated and certified medical devices represents an important challenge for a large-scale use of hypoxic conditioning as a preventive and treatment strategy in various clinical settings.

## Conflict of Interest Statement

The authors declare that the research was conducted in the absence of any commercial or financial relationships that could be construed as a potential conflict of interest.
